# Validation of the Fertility Norms Scale and Association with Fertility Intention and Contraceptive Use in India

**DOI:** 10.1111/sifp.12227

**Published:** 2023-01-23

**Authors:** Nandita Bhan, Nicole E. Johns, Sangeeta Chatterji, Edwin E. Thomas, Namratha Rao, Mohan Ghule, Rebecka Lundgren, Anita Raj

**Affiliations:** Jindal School of Public Health & Human Development, OP Jindal Global University, Sonipat, India.; Center on Gender Equity and Health, University of California San Diego, San Diego, CA, USA.; Center on Gender Equity and Health, University of California San Diego, San Diego, CA, USA.; School of Social & Political Science, The University of Edinburgh, Edinburgh, UK.; Center on Gender Equity and Health, University of California San Diego, San Diego, CA, USA.; Center on Gender Equity and Health, University of California San Diego, San Diego, CA, USA.; Center on Gender Equity and Health, University of California San Diego, San Diego, CA, USA.; Center on Gender Equity and Health, University of California San Diego, San Diego, CA, USA.; Infectious Diseases & Global Public Health, University of California San Diego, San Diego, CA, USA.; Center on Gender Equity and Health, University of California San Diego, San Diego, CA, USA.; Department of Education Studies, School of Social Sciences, University of California San Diego, San Diego, CA, USA.

## Abstract

Social norms related to fertility may be driving pregnancy desire, timing and contraceptive use, but measurement has lagged. We validated a 10-item injunctive Fertility Norms Scale (FNS) and examined its associations with family planning outcomes among 1021 women and 1020 men in India. FNS captured expectations around pronatalism, childbearing early in marriage and community pressure. We assessed reliability and construct validity through Cronbach’s alpha and exploratory factor analysis (EFA) respectively, examining associations with childbearing intention and contraceptive use. FNS demonstrated good reliability (α = 0.65–0.71) and differing sub-constructs by gender. High fertility norm among women was associated with greater likelihood of pregnancy intention [RRR = 2.35 (95% CI: 1.25,4.39); ARRR = 1.53 (95% CI: 0.70,3.30)], lower likelihood of delaying pregnancy [RRR = 0.69 (95% CI: 0.50,0.96); ARRR = 0.72 (95% CI: 0.51,1.02)] and greater ambivalence on delaying pregnancy [RRR = 1.92 (95% CI: 1.18,3.14); ARRR = 1.99 (95% CI: 1.21,3.28)]. Women’s higher FNS scores were also associated with higher sterilization [RRR = 2.17 (95% CI: 1.28,3.66); ARRR = 2.24 (95% CI: 1.32,3.83)], but the reverse was noted for men [RRR = 0.61 (95% CI: 0.36,1.04); ARRR = 0.54 (95% CI: 0.32,0.94)]. FNS indicated better predictive value among women compared to men for key reproductive outcomes. This measure may be useful for social norms-focused evaluations in family planning and warrants cross-contextual study.

## BACKGROUND

Across low- and middle-income countries (LMICs) like India, fertility transitions are well-studied and there has been growing research on social norms related to gender and contraceptive use as important predictors for reproductive health outcomes (Bongaarts 2008; Lerch 2018). Considerable declines in fertility across LMICs have also been noted to be driven by socioeconomic factors and policies of population stabilization (Bryant 2007). India, like these countries, has witnessed sharp aggregate declines in fertility rates to below replacement level (from a total fertility rate [TFR] of 2.2 in 2015–2016 to a TFR of 2.0 in 2019–2020) even as geographic and sociodemographic variations in these rates continue. These declines have been attributed to successes in family planning (FP) programs and multisectoral stakeholder engagements in generating positive messages on family limitation. However, it is also well-recognized that despite these achievements, several structural and social barriers continue to impede community access to sexual and reproductive health services. In particular, India’s contraceptive use patterns continue to demonstrate high reliance on female sterilization post-achievement of desired family size or sex composition, and low or nonuse of contraception early in marriage or spacing between births (IIPS 2015, 2021). These contraceptive use patterns are reflective of continued gaps in our understanding of the drivers of contraceptive use motivations, especially fertility-related norms that may be driving early fertility in marriage, pregnancy intention, and contraceptive use and have remained a neglected area of research(Doepke and Tertilt 2018).

Social norms have been defined as the perception of actual or typical behaviors that hold the standard for an individual or community (Chung and Rimal 2016). Norms are often categorized as “*descriptive norms*,” that is, perception of what is typically done, or as “*injunctive norms*,” that is, perception of what is expected by (significant) others (Chung and Rimal 2016). Adherence to social norms is often driven by the desire to avoid social sanction (e.g., alienation or experience of violence) or to receive positive affirmation (e.g., status, prestige, or support) (Chung and Rimal 2016). Research and theory have increasingly emphasized the role of social norms for health promotion, including for FP programs and in particular to affect contraceptive use, but measurement of norms, and in particular, norms related to fertility and childbearing, have lagged behind the practice (Agha et al. 2021; Beniamino Cislaghi and Heise 2018b; Ben Cislaghi and Heise 2019). The latter can relate to fertility expectations, partner communication on FP, health-seeking behavior, timing and use of contraception, and the number/sex of children. An improved understanding of these norms and the role of family and social influencers can contextualize our understanding of contraceptive use and its determinants (Costenbader, Cislaghi, et al. 2019).

Norms related to fertility draw from gender role expectations of women as mothers (Adams, Salazar, and Lundgren 2013; Schuler, Rottach, and Mukiri 2011; Kane et al. 2016) and reinforce power relations that can undermine women’s reproductive agency and support childbearing coercion from husband or family in ways that impede contraceptive uptake (Solanke 2019). Fertility norms are rooted in social and community expectations around childbearing and thereby are a source of pressure for both women and men. In most contexts, the emphasis of fertility norms is on *pronatalism* (wherein childbearing is viewed as essential to marriage and to family lineage building), and within some contexts, such as India, early in-marriage childbearing and birth to sons (i.e., son preference) (Chabé-Ferret 2019; Khadivzadeh, Latifnejad Roudsari, and Bahrami 2014; Krishnan 2001). Qualitative research from South Asia documents the deeply embedded nature of pronatal attitudes (i.e., expectations that all couples will have children), expectations of early in marriage births as an indicator of a healthy marriage, and son preference as key expectations from newly married couples (Paudel and Acharya 2018; M. Ghule et al. 2015a). Both women and men learn these norms via early in life socialization, and community interactions and in-law as well as natal family pressures to ensure these normative practices ensue soon after marriage (A. Dixit, Averbach, Yore, Kully, Ghule, Battala, Begum, Johns, Vaida, Bharadwaj, et al. 2019; M. Ghule et al. 2015a). Unfortunately, quantitative measurement of these norms continues to be a critical gap in the field (Obermeyer 1996; Jayaraman, Mishra, and Arnold 2009).

There is some evidence of norms based on collective fertility norms, or the aggregate parity in a given geographic area, as well as via social networks, where the behavioral practices of those in the social network serve as the basis of the norm. We see inconsistent findings from these. One study from India found that collective norms indicative of higher fertility were associated with higher fertility for a woman (Mishra and Parasnis 2017), while a study of men in Benin found that normative practices of contraceptive use within a social network had little influence on men’s FP behaviors because men did not discuss those issues with their peers (LeMasters et al. 2021). These findings support the need for research that is contextualized and includes both males and females in this examination. Male authority over fertility and FP decision-making combined with expectations of women taking responsibility for contraceptive use further reinforces the need for research on fertility norms to consider both women and men (Tilahun et al. 2014; Prata et al. 2017). The COVID-19 pandemic further impacted the normative influences as heightened financial insecurity and limited access to health and social support networks led to altered negotiations in contraceptive access and use (Hassan et al. 2022). While new and emerging evidence is bringing to light the role of the pandemic in altering fertility intention and expectations, measurement of norms in the context of the pandemic continues to remain a gap in our field.

This study seeks to address these gaps in our understanding by testing and validating a new measure of fertility norms designed for use with both women and men. Our team developed this measure based on our previous research, evidence reviews, and conversations and guidance from global FP experts (Bhan et al. 2020; M. Ghule et al. 2015a; Costenbader, Cislaghi, et al. 2019; Costenbader et al. 2017). The measure focuses on key aspects of norms, such as pronatalism and early in-marriage childbearing expectations that could predict fertility intention and contraceptive use (M. Ghule et al. 2015a; Nandita Bhan et al. 2020). The measure, which emphasizes injunctive norms and social sanctions, was validated in a rural setting in India during the COVID-19 pandemic, which may have both affected childbearing preferences and access or perceived access to FP services and contraception at the time of the study. We hypothesized that more traditional fertility norms are associated with higher fertility intention and lower reversible contraceptive use. Findings from this work can guide social and behavior change-focused interventions within communities seeking to increase contraceptive uptake.

## METHODS

### Study Design and Participants

We analyzed data from a cross-sectional survey conducted with married couples in Pune district of Maharashtra, India, between February and March 2021. This district is comprised of a rural population of 3.7 million residents across 2,000 villages, with 73.25 percent female literacy and a sex ratio at birth for children born in the last five years of 927 (total) and 786 (rural) females per 1,000 males (IIPS 2015; Census 2011). Junnar, where this study was conducted, is a predominantly rural area (93 percent) with female literacy of 68.7 percent (Census 2011). The state of Maharashtra was at the epicenter of the COVID-19 pandemic in India and Pune district ranked high in terms of COVID-19 cases (Bogam et al. 2022).

The cross-sectional survey involved participants sampled from a two-armed cluster randomized control trial evaluation of an FP and gender equity intervention (CHARM2: Counseling Husbands and Wives to Achieve Reproductive Health and Marital Equity 2), which was aimed at improving contraceptive choice and marital safety (Raj et al. 2022). At the time of recruitment for CHARM2, we enrolled women aged 18–29 years and their husbands. Participants were recruited via household sampling and only include couples where neither partner was sterilized, the couple co-resided in the village for three months and planned to stay for at least two years, and both were fluent in Marathi (local language) (n=1,201) (Anvita Dixit, Averbach, Yore, Kully, Ghule, Battala, Begum, Johns, Vaida, and Bharadwaj 2019). Details on the original study are available elsewhere (Raj et al. 2022). Our research team asked participants of the CHARM2 18-month follow-up survey if they would be open to participation in additional studies in the future, and 90 percent of them agreed to participate in future studies. Approximately six months subsequent to their CHARM2 study completion, we invited those who agreed to further remain in contact with us to participate in a one-time survey on their health and experiences during the COVID-19 pandemic. The period of data collection was nine months after the beginning of the COVID-19 pandemic in India, and we conducted this research in partnership and consultation with local health administration and health centers, following all government-required COVID-19 protection protocols. Of those approached for study participation, 85 percent participated. The final analytic sample included 1,021 married women and 1,020 men for whom fertility norms items were available and who had nonmissing information on all study variables.

### Study Procedure

Our research team contacted all eligible married women and their husbands via household visits between February 1 and March 31, 2021, after the government-imposed lockdown was lifted in the state. Trained masters-level researchers collected all data using electronic tablets; we used sex-matched researchers for all data collection. Women and men were separated at the time of data collection, and both were assured that the researcher would not share responses with anyone outside of the study including their spouses. The survey collected data on a range of health issues, including access to health services, availability of social safety net programs, socioeconomic factors, mental health, and COVID-19 infections. Sociodemographic data on the participants were available through previous demographic data records, thereby ensuring a shorter survey time (approximately 30 minutes). Although we offered the participants the option of survey participation in-person and via telephone, all of them opted for in-person interviews. At the end of the interview, the research staff provided the participants with information regarding available health services as well as COVID-19 relief services available in the study area.

Given the circumstances of the pandemic, the study team followed all state-mandated protocols (e.g., social distancing and mask-wearing) throughout recruitment and data collection. Data were collected in safe outdoor spaces that offered privacy to the participants. Both the researcher and the participant wore a mask at all times, and the research team ensured that the field team and members of the participant household were not showing any symptoms of COVID-19 nor had they had confirmed exposure or positive COVID-19 test. Written informed consent was obtained from the participants prior to data collection and sex-matched data investigators conducted recruitment and interviews. Data were collected using electronic tablets and stored in deidentified formats on password-protected servers. Data quality protocols were followed to ensure data consistency checks and to minimize coding errors.

This study received ethical approval through Sigma Research IRB (#10037/IRB/20–21, November 26, 2020) and the University of California San Diego Human Subjects Committee (#202032, 01/22/21).

### Measurement of Fertility Norms

As noted above, our team developed this measure based on our previous research, evidence reviews, and conversations and guidance from global FP experts (Nandita Bhan et al. 2020; M. Ghule et al. 2015a; Costenbader, Cislaghi, et al. 2019; Costenbader et al. 2017). We emphasized the development of items built from our prior qualitative work from this same study context (M. Ghule et al. 2015a) (Box 1), which showed fertility norms manifesting as (1) pronatal norms or expectations that all married couples must have children; (2) pressure on women and men to demonstrate fertility early in-marriage, with greater pressure on the former; and (3) pressure for a male child. We created a 10-item measure on injunctive fertility norms, including sanctions for nonadherence to normative expectations, for testing in the field. These items were pilot-tested on a sample of 200 women who participated in an FP survey in Uttar Pradesh in November–December 2020 and revised based on feedback received on content, ease of response, and wording to contextualize items. We chose to pilot the items in Uttar Pradesh rather than Maharashtra due to difficulties in timing field site data collection during the COVID-19 pandemic. The pilot test showed good comprehension and completion of items.

Our 10-item *Fertility Norms Scale* (FNS) focused on injunctive norms with items capturing (1) childbearing pressures on women and men soon after marriage; (2) women and men’s experience of sanction or pity if they do not have children at all or within a time frame; and (3) lack of acceptance for couples who have not had children. Item listing is available in Table 1. Responses to these items were coded on a 5-point Likert scale ranging from strongly agree to strongly disagree (coded 1–5). We contextualized items in reference to participant’s definition of their community, which we defined as “*the people you are most often around and those that you may listen to more often*.” We summated items, and those with a higher aggregate score represented more traditional fertility norms, that is, greater normative pressure for childbearing.

### Dependent Variables

We examined associations between fertility norms and key outcomes reported by women in the survey to test for construct validity. Outcomes included: fertility action (taking action to get pregnant or prevent pregnancy), intention to delay pregnancy until after the pandemic, fertility intention (intention for and timing for more children), wife’s use of modern contraception in the past three months, female sterilization at the time of the survey, number of children, and having a living son.

The survey asked all women in the survey if they were *taking action to get pregnant or delay pregnancy*: “Are you currently trying to have a baby or prevent having a baby right now?”. Responses were coded as “I am currently trying to become pregnant,” “I am neither trying to become pregnant nor trying to prevent pregnancy,” and “I am currently trying to prevent pregnancy” (reference).

Women were also asked if they intended to delay pregnancy until after the COVID-19 pandemic. We combined responses for the questions: “would you rather have become pregnant after COVID-19 is over?” asked to pregnant women and “would you like to avoid becoming pregnant until after COVID-19 is over” to nonpregnant women. Evidence of large-scale disruptions have demonstrated the impact of the pandemic on women’s reproductive lives, goals, behaviors, and access to care (Lindberg et al. 2020). However, we expected an interaction of pandemic effects with the influence of strong cultural beliefs and norms. The influence of the latter might also predominate given the lack of clarity, at that time, on how long the pandemic had been likely to last. Responses available included Yes, No, Unsure/Don’t know, and I don’t want to become pregnant again; these were recoded as Yes, Unsure/Don’t know, and No (reference).

To measure fertility intention, the survey asked all women “*Have you decided whether or not to have (more) children?*” (Speizer and Calhoun 2022). Response options included: “Yes, within the next two years,” “Yes, within two or more years,” “Yes, but I am not sure when,” “Don’t know if I want to have (more) children,” and “No I don’t want more children” (reference). The survey assessed among nonpregnant women (n=958) if they had used *modern contraception in the past three months*. Responses were coded as No (reference) versus Yes. The survey also collected data on modern contraceptive use for nonpregnant women by the *type of contraceptive use*, with responses coded as None (reference), Sterilization, and Spacing Methods. The survey asked women if they had *had female sterilization at the time of the survey*, with responses coded as No (reference) versus Yes. We also assessed the number of living children for couples, categorized as none, 1, or 2 and whether they had a living son (No/Yes).

### Statistical Analysis

The sociodemographic characteristics of women and men who participated in the survey are available in Table S1. We assessed key sociodemographic characteristics, including participants’ age, wife’s age at marriage, husband and wife’s education, religion and caste/tribe status, residence in nuclear versus non-nuclear households, and the number of years married. We examined the prevalence of key outcomes in the study, including fertility intention, modern contraceptive use, and the number of children. For the norms measure, we assessed the frequency distributions of items and interitem correlations to understand item clustering and emergent subdimensions. We used exploratory factor analyses (EFAs) to examine scale dimensionality and confirmatory factor analyses using structural equation modeling to examine fit statistics, including the chi-squared value of the model fit, Comparative Fit Index (CFI), and Tucker–Lewis Index (TLI) values. The measure’s internal consistency reliability was assessed using Cronbach’s alpha (benchmarked against a cutoff value of 0.6 demonstrating high reliability) as well as for the measure’s subdimensions (Nunnally 1978). For construct validity, we examined the associations of fertility norms (categorized as a high norm, moderate norm, and low norm for childbearing) with outcomes using logistic and multinomial regression models, reporting odds ratios and relative risk ratios with 95 percent confidence intervals. All analyses were conducted on STATA (v15).

## RESULTS

### Sample Descriptives

Women participants in the survey were on average 26 years of age (mean=25.9 years, 20–32 years of age), while their husbands were 31.6 years of age on average (20–45 years) (Table S1). Nearly, one-third of women and men reported 13 or more years of schooling (women: 31.7 percent, men: 30.05 percent). About one in five women (16.4 percent) reported marriage prior to 18 years of age. Over 90 percent of men and women belonged to the Hindu religion, and over 30 percent belonged to marginalized caste (SC/ST/OBC). Over 87 percent of women reported living in a non-nuclear household, implying a joint or extended family living arrangement.

The prevalence of key outcomes in the study is presented in Table S2. Among nonpregnant women, 61.3 percent reported the use of modern contraception in the past three months. Nearly, 49 percent of nonpregnant women (48.96 percent) reported using modern spacing methods, while 12.3 percent used female sterilization. About 12.3 percent of nonpregnant women reported female sterilization. Nearly, one-third of nonsterilized women reported they intended to have (more) children, with 14.4 percent reporting the intention to have children within the next two years and 16.6 percent reporting the intention to have children in two years or more. About 15 percent of nonsterilized women reported being unsure of the timing for their next child. Over 80 percent of participating nonsterilized women reported, they were currently trying to prevent pregnancy, and one-third (37.8 percent) reported an intention to delay pregnancy until after the COVID pandemic. About 50.6 percent reported having one child and 59.9 percent reported having a son.

### Psychometric Testing of the FNS

Items of the FNS are available in Table 1. These items relate to expectations from newly married couples to have a child soon after marriage, for all married couples to have children and feeling pity for those unable to, and community speaking badly of men and women unable to have a child after more than two years of marriage. Analyses of frequencies showed strong agreement on three items among women–expectation that all married couples have children, pity for those unable to, and considering not having a child as unacceptable. Men also demonstrated agreement with these, along with two additional items: people speaking badly of married women who have not had a child after two or more years of marriage, and community members thinking that there are marital or health problems if a couple has not hada child after two or more years of marriage. Few women and men responded with the option of neither agreeing nor disagreeing; while men did not respond frequently with strong agreement or disagreement. Wide variation in agreement /disagreement was noted, which may also be due to the use of a 5-point Likert scale. Interitem correlations for the FNS demonstrated strong item clusters (Tables S3 and S4). Among women, we found strong correlations between Item 4& Item5 (*speaking badly of married women and men without children*) (r=0.52, p<0.0001); and Item 9 & Item 10 (*common to have bride’s and groom’s parents pressure for children soon after marriage*) (r=0.56, p<0.0001). We also found moderate correlations between Item 2 & Item 3 (*norm to have children* and *pity for couples unable to have children*) (r= 0.29, p<0.001); Item 4 & Item 6 (*speaking badly of married women without children* and *people thinking marital or health problems if no child*) (r=0.39, p<0.0001); and Item 6 & Item 8 (*people thinking marital or health problems if no child* and *asking married couples when they will have children*) (r=0.33, p<0.001). Correlations were similar for the FNS for men. Additionally, we saw a high correlation between Item 8 & Item 9 (*asking married couples when they will have children* and *common to have bride’s parents pressure for children soon after marriage*) (r=0.58, p<0.001). The overall correlation between FNS scores for women and men was low (r=−0.039, p=0.2) and we found low interitem correlations across men and women.

The FNS (ranging between 10 and 50) was normally distributed with values ranging from 15 to 50 among women (mean=30.9) and from 17 to 43 among men (mean=32.4). We used tertiles of fertility norms to quantify low, medium, and high FNS, with higher score values or quantiles indicating greater injunctive norm for childbearing. Among women, the FNS score was higher among younger women (p=0.03), women with secondary or lower education (p=0.0002), and women who married before 18 years of age (p=0.04) and for parity 0–1 (p=0.02) (Table 2). Among men, FNS was higher among those above 30 years of age (p=0.01), with secondary or lower education (p=0.001), and from marginalized caste (p=0.0001).

The overall *α* for the FNS was 0.71 for women and 0.65 for men, showing good internal consistency and reliability (Table 3). EFA demonstrated a potential three-factor structure for the FNS among women, including factor 1: *pronatal norm*–*expecting married couples to necessarily have children* (Items 1, 2, 3, 6, 7, & 8; *α* =0.61), factor 2: *parental pressure for childbearing soon after marriage* (Items 9 & 10; *α* =0.70), and factor 3: *speaking badly or social sanction for couples who have not had early fertility* (Items 4 & 5; *α* =0.67). Item 1 loaded almost equally on factors 1 and 2, but was retained for factor 1 given alpha differences. Correlation matrices for the three factors demonstrated a moderate correlation between factors 1 and 2 (0.328), and low correlations between factors 1 and 3 (0.263) and factors 2 and 3 (0.187). Structural equation modeling to conduct confirmatory factor analyses for the three-factor solution provided chi-square value =129.49 (p<0.0001), (showing the good fit of the model vs. saturated model), RMSEA =0.055 (fit improves as the value approaches 0.01), CFI value=0.942 (values over 0.95 indicate good fit), and TLI value=0.919 (good fit if over 0.9).

For men, item loadings as well as subconstructs differed. We found evidence for the emergence of three potential factors: factor 1: *asking about or pressuring to have children* (Items: 8, 9, & 10; *α* =0.85), factor 2: *community talk if there are no children* (Items 1, 4, 5, & 6; *α* =0.69), and factor 3: *nonacceptance or pity for those who do not have children* (Items 2, 3, & 7; *α* =0.26). Dropping Item 7 (unacceptable not to have children) improved the overall measure alpha for men (*α* =0.67). However, to ensure comparability by gender, we kept the same items for the FNS among men and women, retaining the measure as a unidimensional construct. Differences in subconstructs demonstrate the gendered nature of FNS.

### Fertility Norms and FP Outcomes

Associations of the 10-item FNS scale (full scale) with FP outcomes demonstrate the construct validity of the measure, especially among women (Tables 4a–d). Compared to women who reported low norm, women reporting high FNS were more likely to report that they were trying to get pregnant (RRR =2.35 [95 percent CI: 1.25, 4.39]); adjusting for covariates reduced the effect and led to nonsignificant findings (Table 4a). High FNS among women was associated with a lower likelihood of delaying pregnancy until after the pandemic (RRR=0.69 [95 percent CI: 0.50, 0.96]) and greater ambivalence on delaying pregnancy (ARRR=1.99 [95 percent CI: 1.21, 3.28]). High FNS was also associated with women’s greater ambivalence on the timing of their next child (ARRR=1.72 [95 percent CI: 1.09, 2.70]) (Table 4b). On contraception use outcomes, compared to nonpregnant women who reported low FNS, those who reported moderate FNS were twice as likely to report sterilization (RRR=2.24 [95 percent CI: 1.32, 3.83]) but findings were not statistically significant for women reporting high FNS, adjusted for covariates (Table 4c). High FNS was also associated with having no children (ARRR=3.75 [95 percent CI: 1.72, 8.19]) and with having two children (ARRR=1.47 [95 percent CI: 1.04, 2.10]), but not with having a living son. Among men, high FNS was associated with a lower likelihood of a wife’s sterilization in adjusted models (AOR=0.54 [95 percent CI: 0.32, 0.94]) (Table 4c).

## DISCUSSION

We validated a novel measure of fertility norms that provides insight into expectations and pressures related to childbearing experienced by women and men in rural India. While the FNS was reliable for both women and men, it demonstrated better psychometrics for women compared to men. Among women, we found three subconstructs of fertility norms–pronatalism, pressure for early childbearing, and sanction for not having children early in marriage. In comparison, we found two subconstructs of fertility norms for men–(1) common to be asked about childbearing and (2) community talk if one does not have children early in marriage. Differing subdimensions of norms across women and men provide further evidence of the gendered nature of fertility norms, which has been noted in other contexts, and these differences may be drawing from gender role ideals of femininity and masculinity (Adams, Salazar, and Lundgren 2013; Schuler, Rottach, and Mukiri 2011), where women and men balance differing social pressures for children with issues of economic security (Adams, Salazar, and Lundgren 2013). These gender differences may also be attributed to differential access to or types of social networks and influencers by gender, which may affect both the type and degree of pressure experienced. Research has also been growing on the role of key family influencers, such as mothers-in-law, and community influencers, such as self-help groups in influencing FP choices and behaviors among women. In comparison, we know little of who influences men and what are the motivations behind the influence. In our study, while men reported a greater mean score for FNS compared to women, they also demonstrated a smaller range, with validation psychometrics being more robust for women. This mixed trend reinforces the current evidence where reporting of fertility norms may not translate clearly into predictive contraceptive outcomes.

For women, our study reported evidence for pronatalism and early-in-marriage fertility, which has often in the case of India, translated into small intervals between ages at marriage and first birth (i.e., early in-marriage birth), and a greater likelihood for contraceptives, especially if the previous child born is male. This is also evident given the lack of association between norms and having a living son, reflecting a strong presence of son preference norm and greater value of family limitation once a male child is born. Among men, in comparison, we noted two emergent subconstructs included being asked often about childbearing and facing community talk, which may represent a question to their masculinity. Further investigation on these gendered pathways is needed to understand these different fertility pressures (e.g., women may face social chatter, censure, or violence, while men experience pressure to demonstrate virility in their social circle). Qualitative data from prior research also show the pressure faced from relatives and neighbors on newly married couples to begin having children, but women are more vulnerable to this pressure and the resulting backlash, including from their partners (Adams, Salazar, and Lundgren 2013). While we considered the full 10-item scale for analyses as these subconstructs need further exploration and for reasons of coherence and consistency, we recognize that there is a need and value for deeper engagement by gender on the subconstructs offered by this measure. Exploring these subthemes in order to develop norms-focused interventions that cater to community values and expectations related to childbearing may not only provide respite from the pressure related to childbearing, but may also enable contraception use earlier in marriage and avert unintended pregnancies.

Our findings showed that higher fertility norm reported by women was associated with a greater likelihood of trying to get pregnant, a lower likelihood of delaying pregnancy, and greater ambivalence on pregnancy timing, despite the risks associated with the pandemic and lower access to FP services. Men’s reports of fertility norms were also associated with a lower likelihood of a wife’s sterilization. For men, we did not find statistically significant associations with pregnancy intentions, which may reflect that while there are perceived expectations or community talk regarding childbearing, we did not find that this pressure translates into predicted outcomes. The gendered nature of pressure and its manifestation in marital and fertility decision-making needs further unpacking, and examining this in relation to previous work on reproductive coercion (Silverman and Raj 2014) may be insightful. While the study provides data on women and men, subconstructs demonstrated gender differences in emergent themes, representing different ways in which men and women might be experiencing, internalizing, and expressing pressures related to childbearing.

We validated the scale in a rural community in the state of Maharashtra, a region considered to have a higher standard of living compared to the national average, and that has also reported higher than national average rates of use of modern contraception, especially sterilization. However, despite these more visible rates of community development, previous research has shown that traditional social and cultural norms related to marriage, FP decision-making, and son preference continue to prevail (Mohan Ghule et al. 2015b). Data used in this analysis also come from a survey that was conducted in an area following an FP intervention with couples and this may have also influenced discussion and norm related to fertility and childbearing. The intervention may have led to spousal or community conversation on childbearing, and hence it is possible that our findings represent an underestimate in terms of the association of the FNS to the outcomes. Though notably, the inclusion of treatment condition in the models showed no meaningful effects. While both the context and the intervention may explain the higher interest and use of contraception in the community, the pandemic may also have altered or introduced new kinds of fertility-related pressures. These will become clearer as more research on the short- and long-term impacts of the pandemic on pregnancy intention and contraceptive use comes forward.

However, our findings showed that despite a raging pandemic, individuals who felt fertility pressure were not inclined to delay their next pregnancy and/or remained ambivalent on its timing. These results suggest that FP interventions along with improving contraceptive knowledge and access may also need to shift attitudes and norm-relieving pressures for early childbearing and enhancing agency and choice within marriages. Shifting norms requires sustained multisectoral engagement that goes beyond the individual level and that can be augmented by more structural influences, such as media and policy “nudges.” It also needs wider social and behavior change communication interventions in FP that extend to women and men’s network of “influencers” through diffusion and network-based approaches. An important step in this direction will be to investigate who are the “key” influencers for women and men, differences in their networks as well as in the pathways of influence. The growing work on male and mother-in-law engagement, to understand spoken and unspoken pathways of influence and to design interventions that cater to husbands (Anvita Dixit, Averbach, Yore, Kully, Ghule, Battala, Begum, Johns, Vaida, and Bharadwaj 2019) as well as mothers-in-law may provide some direction. In addition, strategies that enhance women and girls’ agency (e.g., girl child empowerment, education, and prevention of child marriage), male engagement, and spousal communication can also be important in challenging unfavorable gender norms within households.

While the FNS was developed and validated in the Indian context, it has drawn from and contributed to the growing body of knowledge on social norms that has been emerging across contexts (Richardson et al. 2016; Costenbader, Zissette, et al. 2019; Sedlander et al. 2022). These learnings, both in conceptualization and measurement science, are growing due to new and emerging cross-national platforms that have allowed easier collaboration and sharing of measures, as well as associated learnings. Further testing of this measure, within India and outside, is needed to improve its validity, generalizability, and relevance for monitoring and evaluation in FP programming. We found in our study that FNS has relevance for predicting pregnancy intention and contraceptive use, especially for women, and can enable FP programs to deliver counseling and services more effectively in the community.

### Limitations of the Study

Findings from this study need to be seen in the light of *five* limitations/constraints. First, our measure focused on injunctive norms drawing on the participants’ felt or perceived expectations of childbearing or what was expected of them. While this provided us valuable insight, we did not have data related to descriptive fertility norms that could provide us insight into participants’ understanding of what their network actually does. An understanding of both descriptive and injunctive norms may collectively provide a more comprehensive understanding of normative influences and shed light on their complementarities as well as their relative significance for women and men (Sedlander et al. 2022).

Second, in the study, participants were asked to define their “community” following which items on fertility norms were administered. This approach has many advantages, as it allows the participants to conceptualize their own community and the researcher does not make assumptions regarding key influencers. However, we do recognize that this approach to defining the community can be dynamic, vary by gender, age, and social markers (caste, wealth, and education), and may create ambiguity in interpreting who is setting fertility-related expectations. As research on influencers and understanding social networks of young married women and men is still limited, the former approach was considered more suited for the study, as it allowed the participants to describe their perceived expectations freely without bounding them to responses in relation to an influencer who may or may not be relevant for them. Future research that furthers our understanding of the social networks of young married women and men can improve how we contextualize this “community” for new and emerging measures of norms.

Third, the measure’s differential performance across men and women opens doors to future research engaging with a gendered understanding of fertility expectations and pressures. These likely draw from our understanding of masculinity, gender power relations, and cultural connotations of fertility, in light of intergenerational value changes. FP programs need to engage with male engagement strategies directly within counseling and programs using evidence from existing and emerging high-impact practices (High-Impact Practices in Family Planning 2018), as well as meaningfully engage other key influencers. For instance, our recent work shows the important role of the mother-in-law for women (N. Bhan et al. 2022), but we do not fully understand the pathway to influence or its variability. As male engagement and norms-focused interventions develop, there will be a need to engage with these questions within FP programs, and to direct services and programs not only toward women, but also to identify, leverage, and channel interventions toward other key influencers.

Fourth, we found a wide variation in the agreement/disagreement frequencies reflecting either social desirability bias or extreme views in how the community understood and responded to these questions. The use of a 5-point Likert scale, a standard in the field, was also tested in this context. In future research, we would like to explore how these emergent themes could be developed into subscales that might explain the gendered nuances of fertility norms more effectively, as well as whether or not a 3-point Likert scale could provide a more cohesive picture of the responses.

Finally, while subdimensions of our norms measure indicated emergent themes, such as community talk, sanctions, and backlash, we did not have adequate data to explore these aspects which may be critical to understanding how women and men exercise their reproductive agency to access FP services. These subthemes can be valuable additions to understanding gender and power within and outside the household with implications for accessing FP programs and services, and need to be further interrogated in FP monitoring and evaluation surveys to understand their channels of influence.

## CONCLUSION

We validated a 10-item injunctive FNS that showed good reliability and validity for women and men in India. The FNS assessed norms and pressures for early childbearing and can be a useful tool for FP programs to improve the understanding of fertility intention and contraceptive use among newly married and low parity women. We found evidence of gender differences in emergent themes in fertility norms and recommend further testing and validation across other contexts.

## Supplementary Material

TABLE S1 Sociodemographic characteristics of women and their husbands in the survey in Maharashtra, India TABLE S2 Key family planning outcomes in the study in Maharashtra, India TABLE S3 Intercorrelations of Items of the Fertility Norms Scale for women in Maharashtra, India TABLE S4 Intercorrelations of Items of the Fertility Norms Scale for men in Maharashtra, India

## Figures and Tables

**TABLE 1:** Frequency distribution of items of the Fertility Norms Scale among women and their husbands in the study in Maharashtra, India

	a. Women (n=1,021)					b. Men (n=1,020)				
		
	i. Strongly agree	ii. Agree	iii. Neither agree nor disagree	iv. Disagree	v. Strongly disagree	i. Strongly agree	ii. Agree	iii. Neither agree nor disagree	iv. Disagree	v. Strongly disagree

a. In my community, it is expected for newly married couples to have a child soon after marriage. (AW5A)	69 (6.73)	300 (29.27)	2 (0.2)	601 (58.63)	53 (5.17)	20 (1.95)	416 (40.55)	73 (7.12)	506 (49.32)	11 (1.07)
b. In my community, it is expected for all married couples to have children. (AW5B)	299 (29.17)	676 (65.95)	0	50 (4.88)	0	85 (8.28)	823 (80.21)	45 (4.39)	72 (7.02)	1 (0.10)
c. In my community, people feel a lot of pity for married couples who are unable to have children. (AW5C)	258 (25.17)	702 (68.49)	2 (0.2)	61 (5.95)	2 (0.2)	48 (4.68)	811 (79.04)	49 (4.78)	117 (11.40)	1 (0.10)
d. In my community, people speak badly of married women who have not had a child after two or more years of marriage. (AW5D)	79 (7.71)	422 (41.17)	7 (0.68)	475 (46.34)	42 (4.10)	57 (5.56)	663 (64.62)	28 (2.73)	272 (26.51)	6 (0.58)
e.In my community, people speak badly of married men who have not had a child after two or more years of marriage. (AW5E)	20 (1.95)	165 (16.10)	11 (1.07)	752 (73.37)	77 (7.51)	12 (1.17)	562 (54.78)	76 (7.41)	370 (36.06)	6 (0.58)
f. In my community, people will think there are marital or health problems if a couple has not had a child after two or more years of marriage. (AW5F)	112 (10.93)	489 (47.71)	29 (2.83)	376 (36.68)	19 (1.85)	77 (7.50)	655 (63.84)	76 (7.41)	213 (20.76)	5 (0.49)
g. In my community, it is unacceptable for couples to choose not to have children. (AW5G)	176 (17.17)	696 (67.90)	3 (0.29)	147 (14.34)	3 (0.29)	59 (5.75)	573 (55.85)	52 (5.07)	330 (32.16)	12 (1.17)
h. In my community, it is common to ask married couples without a child when they will have children. (AW5H)	92 (8.98)	480 (46.83)	1 (0.10)	420 (40.98)	32 (3.12)	14 (1.36)	467 (45.52)	39 (3.80)	447 (43.57)	59 (5.75)
i. In my community, it is common for parents of the bride to pressure couples to have children soon after marriage. (AW5I)	5 (0.49)	61 (5.95)	3 (0.29)	738 (72.0)	218 (21.27)	3 (0.29)	302 (29.43)	52 (5.07)	583 (56.82)	86 (8.38)
j. In my community, it is common for parents of the groom to pressure couples to have children soon after marriage. (AW5J)	9 (0.88)	191 (18.63)	2 (0.20)	662 (64.59)	161 (15.71)	19 (1.85)	403 (39.28)	33 (3.22)	486 (47.37)	85 (8.28)

**TABLE 2:** Mean differences and associations of the Fertility Norms Score with key covariates for women and men

	a. n	b. Mean Fertility Norm Score (95% CI)	c. T-statistic (p value)

**A. Women**			
*a. Age*			
i. 20–25	454	31.27 (30.80, 31.74)	2.11 **(p=0.03)**
ii. 26–31	571	30.60 (30.18, 31.01)	
*b. Education*			
i. Secondary or less	425	31.59 (31.11, 32.07)	3.69 **(p=0.0002)**
ii. Higher secondary+	600	30.41 (30.00, 30.81)	
*c. Caste*			
i. SC/ST/OBC	304	30.78 (30.21, 31.36)	−0.45 (p=0.6)
ii. General	721	30.94 (30.57, 31.31)	
*d. Religion*			
i. Hindu	947	30.81 (30.48, 31.14)	−1.87 (p=0.06)
ii. Non-Hindu	78	31.93 (30.93, 32.93)	
*e. Child marriage*			
i. No	857	30.75 (30.42, 31.09)	−2.01 **(p=0.04)**
ii. Yes	168	31.62 (30.80, 32.44)	
*f. Number of children*			
i. 0–1	572	30.59 (30.16, 31.02)	−2.18 **(p=0.02)**
ii. 2–3+	453	31.29 (30.83, 31.74)	
*g. Nuclear household*			
i. No	892	30.81 (30.47, 31.14)	−1.4 (p=0.1)
ii. Yes	133	31.50 (30.59, 32.41)	
**B. Men**			
*a. Age*			
i. 20–30	887	32.32 (32.01, 32.63)	−2.3 **(p=0.01)**
ii. 30–45	139	33.32 (32.52, 34.12)	
*b. Education*			
i. Secondary or less	447	32.97 (32.56, 33.39)	3.12 **(p=0.001)**
ii. Higher secondary+	579	32.05 (31.66, 32.45)	
*c. Caste*			
i. SC/ST/OBC	312	33.34 (32.81, 33.87)	4.01 **(p=0.0001)**
ii. General	714	32.07 (31.73, 32.41)	
*d. Religion*			
i. Hindu	947	32.46 (32.16, 32.76)	0.10 (p=0.9)
ii. Non-Hindu	79	32.40 (31.46, 33.34)	
*e. Nuclear household*			
i. No	891	32.36 (32.05, 32.67)	−1.6 (p=0.1)
ii. Yes	135	33.07 (32.31, 33.82)	
*f. Number of children*			
i. 0–1	574	32.63 (32.23, 33.02)	1.34 (p=0.1)
ii. 2–3+	452	32.23 (31.81, 32.65)	

**TABLE 3:** Factor loadings from the rotated matrix and reliability of the Fertility Norms Scale for women and men in Maharashtra, India

	a. Women				b. Men			
		
In my community,	1. One-factor model	2. Three-factor models (Varimax Rotation)			1. One-factor model	2. Three-factor models (Varimax Rotation)		

a. it is expected for newly married couples to have a child soon after marriage.	0.4329	0.3902		0.4897	0.4809		0.4804	
b. it is expected for all married couples to have children.	0.4207	0.7018			0.1640			0.7100
c. people feel a lot of pity for married couples who are unable to have children.	0.5373	0.6429			0.0940			0.6626
d. people speak badly of married women who have not had a child after two or more years of marriage.	0.6978			0.7582	0.4976		0.8324	
e. people speak badly of married men who have not had a child after two or more years of marriage.	0.5303			0.8540	0.3641		0.8535	
f. people will think there are marital or health problems if a couple has not had a child after two or more years of marriage.	0.6649	0.5572		0.3417	0.3981		0.6934	
g. it is unacceptable for couples to choose not to have children.	0.4598	0.4756	0.3305		0.1762			0.5350
h. it is common to ask married couples without a child when they will have children.	0.5081	0.5567			0.7172	0.8088		
i. it is common for parents of the bride to pressure couples to have children soon after marriage.	0.5288		0.8270		0.8002	0.8921		
j. it is common for parents of the groom to pressure couples to have children soon after marriage.	0.5382		0.8183		0.7972	0.9142		
Cronbach’s alpha value	0.7176	0.6095	0.6981	0.6697	0.6522	0.8588	0.6988	0.2658
# of items	10	6	2	2	10	3	4	3
Average interitem correlation	0.1857	0.1925	0.3799	0.5423	0.1438	0.7639	0.3416	0.0704

**TABLE 4a:** a Associations of women and men’s reported Fertility Norm Scale with action to delay fertility among women in Maharashtra, India

	*a. Taking action to get pregnant or delay pregnancy* (reference: Trying to prevent pregnancy)				b. Delaying pregnancy until after COVID (reference: No)			
		
	*i. Trying to become pregnant*		*ii. Neither prevent not trying (ambivalence)*		i. Yes:		ii. Unsure:	
				
	RRR	ARRR	RRR	ARRR	RRR	ARRR	RRR	ARRR

a. Women’s Fertility Norm (Ref: Low)								
i. Moderate	1.15 (0.57, 2.33)	1.13 (0.51, 2.51)	0.66 (0.35, 1.23)	0.55 (0.29, 1.07)	0.73 (0.53, 1.002)	0.71* (0.51, 0.99)	1.38 (0.83, 2.30)	1.38 (0.82, 2.31)
ii. High	2.35* (1.25, 4.39)	1.53 (0.70, 3.30)	0.91 (0.50, 1.64)	0.71 (0.38, 1.32)	0.69* (0.50, 0.96)	0.72 (0.51, 1.02)	1.92* (1.18, 3.14)	1.99* (1.21, 3.28)
b. Men’s Fertility Norm (Ref: Low)								
i. Moderate	1.19 (0.63, 2.23)	1.10 (0.51, 2.35)	0.93 (0.49, 1.76)	0.87 (0.46, 1.67)	1.16 (0.84, 1.61)	1.20 (0.85, 1.70)	0.89 (0.56, 1.42)	0.91 (0.57, 1.46)
ii. High	1.15 (0.62, 2.14)	0.66 (0.31, 1.43)	1.19 (0.66, 2.16)	0.99 (0.53, 1.83)	1.04 (0.75, 1.43)	1.11 (0.79, 1.56)	0.65 (0.40, 1.05)	0.66 (0.40, 1.07)

Note: Adjusted for age, education, religion, caste, living in a nuclear household, and number of years married.

**TABLE 4b:** Associations of women and men’s reported Fertility Norm Scale with fertility intention for women in Maharashtra, India

	Decided whether or not to have children (reference: No I don’t want more children)							
	
	*a. Yes within next two years or more:*		*b. Yes, within two years:*		*c. Yes, but unsure time*		*d. Don’t know if want more children:*	
				
	RRR	ARRR	RRR	ARRR	RRR	ARRR	RRR	ARRR

**a. Women’s reported Fertility Norm** (Ref: Low)	1.00	1.00	1.00	1.00	1.00	1.00	1.00	1.00
i. Moderate	0.90 (0.57, 1.43)	0.81 (0.50, 1.30)	0.68 (0.45, 1.03)	0.59* (0.38, 0.93)	0.84 (0.52, 1.34)	0.83 (0.51, 1.35)	1.54 (0.67, 3.53)	1.70 (0.73, 3.96)
ii. High	1.29 (0.83, 2.00)	1.28 (0.80, 2.04)	0.77 (0.50, 1.18)	0.82 (0.52, 1.30)	1.55* (1.004, 2.39)	1.72* (1.09, 2.70)	0.60 (0.20, 1.82)	0.62 (0.20, 1.89)
**b. Men’s reported Fertility Norm** (Ref: Low)	1.00							
i. Moderate	1.19 (0.75, 1.89)	1.12 (0.69, 1.81)	0.95 (0.62, 1.47)	0.95 (0.59, 1.51)	0.95 (0.62, 1.47)	0.94 (0.60, 1.47)	0.94 (0.36, 2.43)	0.91 (0.35, 2.39)
ii. High	1.38 (0.88, 2.15)	1.34 (0.84, 2.13)	1.12 (0.75, 1.70)	1.23 (0.78, 1.92)	0.72 (0.46, 1.13)	0.73 (0.45, 1.16)	1.33 (0.56, 3.16)	1.34 (0.55, 3.24)

Note: Adjusted for age, education, religion, caste, living in a nuclear household, and number of years married.

**TABLE 4c:** Associations of women and men’s reported Fertility Norm Scale with contraceptive use for women in Maharashtra, India

			b. Wife used modern contraception in the past three months by type (reference: None: only non-pregnant women)					
			
	a. Wife used modem contraception in the past three months (only nonpregnant women):		a. Sterilization		b. Spacing Methods		c. Wife had undergone sterilization at the time of survey: Yes	

a. Women’s Fertility Norm (Ref: Low)	OR	AOR	RRR	ARRR	RRR	ARRR	OR	AOR
i. Moderate	1.36 (0.98, 1.87)	1.35 (0.98, 1.87)	2.17* (1.28, 3.66)	2.24* (1.32, 3.83)	1.23 (0.88, 1.71)	1.20 (0.86, 1.69)	1.93* (1.19, 3.13)	2.02* (1.23, 3.31)
ii. High	0.83 (0.60, 1.13)	0.82 (0.59, 1.13)	1.37 (0.80, 2.33)	1.26 (0.73, 2.16)	0.73 (0.53, 1.02)	0.74 (0.53, 1.04)	1.62 (0.98, 2.66)	1.51 (0.90, 2.52)
b. Men’s Fertility Norm (Ref: Low)								
i. Moderate	0.88 (0.64, 1.21)	0.88 (0.64, 1.22)	0.87 (0.53, 1.41)	0.78 (0.47, 1.28)	0.88 (0.63, 1.24)	0.90 (0.64, 1.28)	0.91 (0.58, 1.43)	0.81 (0.51, 1.29)
ii. High	1.03 (0.75, 1.41)	1.05 (0.76, 1.45)	0.61 (0.36, 1.04)	0.54* (0.32, 0.94)	1.15 (0.83, 1.60)	1.23 (0.87, 1.72)	0.57* (0.35, 0.93)	0.49* (0.29, 0.83)

Note: Adjusted odds ratio control for age, education, religion, caste, living in a nuclear household, and number of years married.

**TABLE 4d:** Associations of women and men’s reported Fertility Norm Scale with number of children and having a living son in Maharashtra, India

	Number of children (reference: one child)					
		
	a. No child		b. Having two children		Has a living son: Yes	

a. Women’s Fertility Norm (Ref: Low)	RRR	ARRR	RRR	ARRR	RRR	ARRR
i. Moderate	1.40 (0.62, 3.17)	1.22 (0.52, 2.86)	1.36* (1.005, 1.85)	1.54* (1.08, 2.17)	1.21 (0.89, 1.64)	1.25 (0.92, 1.70)
ii. High	3.43* (1.69, 6.97)	3.75* (1.72, 8.19)	1.49* (1.09, 2.03)	1.47* (1.04, 2.10)	0.97 (0.72, 1.32)	0.98 (0.72, 1.34)
**b. Men’s Fertility Norm** (Ref: Low)						
i. Moderate	1.48 (0.71, 3.11)	1.45 (0.66, 3.18)	1.07 (0.78, 1.45)	1.05 (0.75, 1.49)	0.94 (0.69, 1.27)	0.93 (0.68, 1.28)
ii. High	1.62 (0.80, 3.27)	2.00 (0.94, 4.25)	0.84 (0.62, 1.14)	0.74 (0.53, 1.05)	0.84 (0.62, 1.13)	0.84 (0.61, 1.14)

Note: Adjusted for age, education, religion, caste, living in a nuclear household, and number of years married.

## Data Availability

Data used for presented analyses and analytic code are available upon reasonable request from the corresponding author.

## Box 1: Illustrations of fertility pressures as expressed in the community (M. Ghule et al. 2015a; Mohan Ghule et al. 2015b)

“There is pressure from elders in the family. If we don’t have child soon after marriage, then they will speak badly about the wife. They will consider it as her fault and taunt her. The[y] will think that she is infertile.” (Husband, Age 23)

“Many times if the couple doesn’t have child after the marriage of one year, then others feel that there must be a problem between the husband and wife.” (Mother-In-Law, Age 60)

“If the couple doesn’t have children after 4–5 years of marriage then we also feel that they should have children. We tell them to go to the doctors or find out whether there is any effect of devil. There is superstition among people.” (Mother-In-Law, Age 40)

“The number of children is decided after the birth of the first child. It only depends upon whether the first child is a son or a daughter.” (Husband, Age 29)
