# Supplementary material for: Validation of the Fertility Norms Scale and Association with Fertility Intention and Contraceptive Use in India
Source: Stud Fam Plann. Author manuscript; Available in PMC 2024 Jun 4. (PMC11147959; doi:10.1111/sifp.12227)
Supplement: TABLE S1 Sociodemographic characteristics of women and their husbands in the survey in Maharashtra, India TABLE S2 Key family planning outcomes in the study in Maharashtra, India TABLE S3 Intercorrelations of Items of the Fertility Norms Scale for women in Maharashtra, India TABLE S4 Intercorrelatio [file NIHMS1993040-supplement-TABLE_S1_Sociodemographic_characteristics_of_women_and_their_husbands_in_the_survey_in_Maharashtra__India__TABLE_S2_Key_family_planning_outcomes_in_the_study_in_Maharashtra__India__TABLE_S3_Intercorrelations_of_Items_o.docx]

**SUPPLEMENTARY TABLES**

TABLE 1 **Sociodemographic Characteristics of Women and their Husbands in the Survey in Maharashtra, India**

|  | **Women (N=1021)** | **Men (N=1020)** |
| --- | --- | --- |
| **a. Age of respondents at survey (in years)** | 25.9 years (20- 31 years) | 31.6 years (20-45 years) |
|  |  |  |
| **b. Education Level (highest)** |  |  |
| i. Primary or no education (0-8) | 127 (12.39) | 135 (13.17) |
| ii. Secondary (9-10) | 298 (29.07) | 311 (30.34) |
| iii. Higher (11-12) | 275 (26.83) | 271 (26.44) |
| iv. Post-secondary (13+) | 325 (31.71) | 308 (30.05) |
|  |  |  |
| **c. Wife age at marriage (in years)** | 19.4 (2.26) (13-27) | == |
| **d. Wife married <18 (Y/N)** |  |  |
| i. No | 857 (83.61) |  |
| ii. Yes | 168 (16.39) |  |
|  |  |  |
| **5. Caste** |  |  |
| i. Scheduled caste | 47 (4.59) | 79 (7.71) |
| ii. Scheduled tribe | 36 (3.51) | 68 (6.63) |
| iii. OBC | 221 (21.56) | 165 (16.10) |
| iv. None/Other | 721 (70.34) | 713 (69.56) |
|  |  |  |
| **6. Religion** |  |  |
| i. Hindu | 947 (92.39) | 946 (92.29) |
| ii. Others (Muslim, Buddhist, Jain, Christian, Other) | 78 (7.61) | 79 (7.71) |
|  |  |  |
| **7. Nuclear household indicator (Y/N)** |  |  |
| i. No | 892 (87.02) |  |
| ii. Yes | 133 (12.98) |  |
|  |  |  |
| **8. Wife reports number of living children** |  |  |
| i. 0 | 53 (5.17) |  |
| ii. 1 | 519 (50.63) |  |
| iii. 2 | 391 (38.15) |  |
| iv. 3+ | 62 (6.05) |  |
|  |  |  |
| **9. Has living sons** |  |  |
| i. No | 411 (40.10) |  |
| ii. Yes | 614 (59.90) |  |
|  |  |  |
| **10. Has living daughters** |  |  |
| i. No | 446 (43.51) |  |
| ii. Yes | 579 (56.49) |  |
|  |  |  |

TABLE 2 **Key Family Planning Outcomes in the Study in Maharashtra, India**

| **Key Dependent Variables** | N (%) |
| --- | --- |
| ***a. Are you currently trying to have a baby or prevent having a baby right now? (n=955)*** |  |
| i. Currently trying to prevent becoming pregnant | 825 (80.36) |
| ii. Currently trying to become pregnant | 64 (6.26) |
| iii. Neither trying to become or prevent pregnancy | 66 (6.91) |
|  |  |
| ***b. Intention to delay pregnancy until after COVID**** |  |
| i. Yes | 386 (37.81) |
| ii. No | 507 (49.66) |
| iii. Unsure/Don’t know | 128 (12.54) |
|  |  |
| ***c. Have you decided whether or not to have (more) children?*** |  |
| i. Yes (< 2 years) | 147 (14.38) |
| ii. Yes (>2+ years) | 170 (16.63) |
| iii. Yes unsure when | 148 (14.48) |
| iv. No I don’t want more (include a number of currently pregnant) | 527 (51.57) |
| v. Don’t know if I want more children | 30 (54.50) |
|  |  |
| **d. Wife used modern contraception in past 3 months** (only non-pregnant women): (n=958) |  |
| i. No | 371 (38.73) |
| ii. Yes | 587 (61.27) |
|  |  |
| ***e. Wife used modern contraception in past 3 months (only non-pregnant women) by type*** (n=958) |  |
| i. No | 371 (38.73) |
| ii. Sterilization | 118 (12.32) |
| iii. Spacing Methods | 469 (48.96) |
|  |  |
| ***f. Wife had female sterilization at Covid FU*** |  |
| i. No | 907 (88.49) |
| ii. Yes | 118 (11.51) |
|  |  |
| ***g. Used Spacing Methods*** |  |
| i. No (including pregnant, got sterilized) | 556 (54.24) |
| ii. Yes | 469 (45.76) |
|  |  |
| ***h. Number of living children*** |  |
| i. None | 53 (5.17) |
| ii. 1 | 519 (50.63) |
| iii. 2+ | 453 (44.20) |
|  |  |
| ***i. Having a living son*** |  |
| i. No | 411 (40.10) |
| ii. Yes | 614 (59.90) |

*For intention to delay pregnancy until COVID-19 pandemic, we combined responses for the questions: “*would you rather have become pregnant after COVID-19 is over*?” asked to pregnant women and “*would you like to avoid becoming pregnant until after COVID-19 is over*” to non-pregnant women. Final responses, available as Yes, No, Unsure/Don’t know, and I don’t want to become pregnant again were recoded as Yes, Unsure/Don’t know, and No (reference).

TABLE 3 **Inter-correlations of Items of the Fertility Norms Scale for Women in Maharashtra India**

|  | AW5A | AW5B | AW5C | AW5D | AW5E | AW5F | AW5G | AW5H | AW5I | AW5J |
| --- | --- | --- | --- | --- | --- | --- | --- | --- | --- | --- |
| a. Newly married couples have child soon after marriage. (AW5A) | 1.000 | --- | --- | --- | --- | --- | --- | --- | --- | --- |
| b. All married couples have children. (AW5B) | 0.2467 | 1.000 | --- | --- | --- | --- | --- | --- | --- | --- |
| c. People feel pity for married couples unable to have children. (AW5C) | 0.2284 | 0.2966 | 1.000 | --- | --- | --- | --- | --- | --- | --- |
| d. People speak badly of married women without child after 2+ years of marriage. (AW5D) | 0.2331 | 0.1278 | 0.2553 | 1.000 | --- | --- | --- | --- | --- | --- |
| e. People speak badly of married men without child after 2+ years of marriage.  (AW5E) | 0.2282 | 0.0829 | 0.1540 | 0.5202 | 1.000 | --- | --- | --- | --- | --- |
| f. People think marital or health problems if no child after 2+ years of marriage. (AW5F) | 0.2347 | 0.2699 | 0.2756 | 0.3982 | 0.2243 | 1.000 | --- | --- | --- | --- |
| g. Unacceptable to not have children. (AW5G) | 0.0996 | 0.1692 | 0.2140 | 0.1927 | 0.1050 | 0.2275 | 1.000 | --- | --- | --- |
| h. Common to ask married couples when they will have children. (AW5H) | 0.1128 | 0.1742 | 0.2365 | 0.2443 | 0.0840 | 0.3395 | 0.1853 | 1.000 | --- | --- |
| i. Common for bride’s parents to pressure for children soon after marriage. (AW5I) | 0.0399* | 0.0543* | 0.1341 | 0.2417 | 0.1762 | 0.2120 | 0.1871 | 0.1998 | 1.000 | --- |
| j. Common for groom’s parents to pressure for children soon after marriage. (AW5J) | 0.0614 | 0.0492* | 0.1225 | 0.2867 | 0.1787 | 0.2144 | 0.2059 | 0.1593 | 0.5631 | 1.000 |

All correlations significant at 0.05 level of significance, except the ones in *

TABLE 4 **Inter-correlations of Items of the Fertility Norms Scale for Men in Maharashtra India**

|  | AW5A | AW5B | AW5C | AW5D | AW5E | AW5F | AW5G | AW5H | AW5I | AW5J |
| --- | --- | --- | --- | --- | --- | --- | --- | --- | --- | --- |
| a. Newly married couples have child soon after marriage. (AW5A) | 1.000 | --- | --- | --- | --- | --- | --- | --- | --- | --- |
| b. All married couples have children. (AW5B) | 0.1512 | 1.000 | --- | --- | --- | --- | --- | --- | --- | --- |
| c. People feel pity for married couples unable to have children. (AW5C) | -0.0282* | 0.1942 | 1.000 | --- | --- | --- | --- | --- | --- | --- |
| d. People speak badly of married women without child after 2+ years of marriage. (AW5D) | 0.2751 | 0.0198* | 0.0432* | 1.000 | --- | --- | --- | --- | --- | --- |
| e. People speak badly of married men without child after 2+ years of marriage.  (AW5E) | 0.2170 | 0.0241* | 0.1150 | 0.6961 | 1.000 | --- | --- | --- | --- | --- |
| f. People think marital or health problems if no child after 2+ years of marriage. (AW5F) | 0.2599 | -0.0466* | 0.0807* | 0.3708 | 0.3979 | 1.000 | --- | --- | --- | --- |
| g. Unacceptable to not have children. (AW5G) | -0.0372* | 0.1063 | 0.0738 | 0.1049 | -0.0363* | -0.1293 | 1.000 | --- | --- | --- |
| h. Common to ask married couples when they will have children. (AW5H) | 0.1915 | 0.0590* | -0.0180* | 0.1157 | -0.0187* | 0.0944 | 0.1407 | 1.000 | --- | --- |
| i. Common for bride’s parents to pressure for children soon after marriage. (AW5I) | 0.2066 | 0.0627 | 0.0425* | 0.0870 | 0.0228* | 0.1331 | 0.0715 | 0.5803 | 1.000 | --- |
| j. Common for groom’s parents to pressure for children soon after marriage. (AW5J) | 0.1815 | 0.0809 | 0.0063* | 0.1228 | -0.0374* | 0.0526* | 0.1688 | 0.6140 | 0.8216 | 1.000 |

All correlations significant at 0.05 level of significance, except the ones in *
